# Systemic autoimmune disease in asbestosis rapidly responding to anti-interleukin-1beta antibody canakinumab: a case report

**DOI:** 10.1186/s12891-015-0602-6

**Published:** 2015-06-14

**Authors:** Laura Niccoli, Emanuele Cassarà, Olga Kaloudi, Carlotta Nannini, Micaela Romagnoli, Fabrizio Cantini

**Affiliations:** Consultant in Rheumatology, Rheumatology Division, Hospital of Prato, Prato, Italy; Consultant in Pneumology, Pneumologie et Addictologie CHU Arnaud de Villeneuve 191, Av. du Doyen Gaston Giraud, 34295 Montpellier, France

**Keywords:** Asbestosis, Interleukin-1beta, Autoimmunity, Canakinumab

## Abstract

**Background:**

Asbestosis is characterized by lung and pleural fibrosis and by immune system dysregulation, with autoantibody production and systemic immune-mediated disease. No specific therapies are available for asbestosis. Recently, the pivotal pathogenic role exerted by interleukin-1beta has been recently reported.

**Case presentation:**

We treated with anti-interleukin 1 beta targeted antibody canakinumab a 67 year old man with asbestosis and long lasting systemic autoimmune features. A dramatic improvement in clinical manifestations was observed at 1 week after the first injection, with complete clinical remission at 4 months.

**Conclusion:**

This case suggests new perspectives for the treatment of asbestosis and its systemic features.

## Background

Asbestosis is a progressive interstitial lung disease caused by inhalation of asbestos fibers occurring in subjects with prolonged exposure to asbestos dust, such as miners, quarrymen, millers, workers of asbestos textiles and insulators. Clinically, the lung involvement is characterized by the development of bilateral diffuse interstitial fibrosis, more pronounced in the lower lobes, and pleural thickening, leading to shortness of breath and dry cough [1]. Although the radiological features of asbestosis are not specific, high-resolution computed tomography (HRCT) usually shows pathognomonic lesions including subpleural fibrosis and pleural plaques [2].

Although not definitively clarified, it is generally accepted that the pathogenesis of pulmonary interstitial inflammation and fibrosis is related to immune mechanisms induced by asbestos [1]. Among pneumoconioses, silicosis represents the most frequent condition inducing systemic autoimmune disorders [3]. However, also asbestosis is known to be associated with serum antinuclear antibody (ANA), rheumatoid factor (RF) positivity [4], and may be complicated by autoimmune diseases such as systemic lupus erythematosus (SLE), systemic sclerosis and rheumatoid arthritis (RA) [5–7].

In absence of specific treatment for asbestosis [2], corticosteroids may represent the only therapy that controls the symptoms related to the associated systemic autoimmune disease.

Recently, an important role of interleukin-1beta (IL-1beta) in the pathogenesis of asbestosis and its systemic autoimmune manifestations has been reported [8]. Indeed, asbestos fibers seem to enhance the release of IL-1beta by alveolar macrophages through the dysregulation of the cellular pool of anti-oxidant thioredoxin and thioredoxin-interacting protein, with the consequent activation of the NALP3 inflammasome, which, in turn, stimulates the expression of the pro-inflammatory cytokine IL-1beta by macrophages [9, 10]. The same crucial role of the NALP3 inflammasome has been demonstrated in the pathogenesis of silicosis [10]. These findings may offer the rationale to treat both the pulmonary and systemic inflammatory process of asbestosis with anti-IL-1beta targeted therapy.

We describe herein the case of a patient with mild asbestosis and systemic autoimmune manifestations successfully treated with canakinumab, an anti-IL-1beta targeted antibody.

## Case presentation

A 67-year old male presented in May 2014 with a 12-year history of low-grade fever, symmetric arthralgia of shoulders, wrists, metacarpo-phalangeal joints and knees, with sporadic episodes of mild joint swelling. He had worked for 35 years as quarryman in different Italian mines and over the last 2-3 years has complained of sporadic dry cough and dyspnea on intense exertion. During the last 12 years, based on a variety of articular and systemic manifestations, persistently elevated erythrocyte sedimentation rate (ESR) and C-reactive protein (CRP), antinuclear antibody (ANA) positivity and, on some occasions, low-titer RF positivity, he had received different diagnoses including RA, SLE and undifferentiated connective tissue disease (UCTD). Over this period, the patient was treated with prednisone with good response, but symptoms flared when the dose was reduced to 5 mg/day. Every attempt to treat the patient with traditional disease modifying anti-rheumatic drugs such as hydroxychloroquine and methotrexate, was unsuccessful, and over time he developed osteoporosis complicated by vertebral fractures, and diabetes.

At the first visit to our center, we recommended tapering and discontinuing the corticosteroids over 2 weeks. After one month, he was febrile (38.4 °C), and had tenderness of wrists, hand joints, and knees, and mild arthritis of the right ankle. Bilateral crackles were detectable on pulmonary auscultation, with otherwise normal physical findings.

Laboratory tests disclosed a normal blood cell count and differential, normal liver and kidney function, ESR 56 mm/h, CRP 7.75 mg/dl (nephelometry; normal value <0.5 mg/dl), and ANA 1/1280, with negative anti-double-stranded DNA antibodies and extractable nuclear antigens, C3 complement fraction levels of 199 mg/dl (normal value 90-180 mg/dl), C4 complement fraction 39 mg/dl (normal value 10-40 mg/dl), and negative RF, anti-cyclic citrullinated peptide antibody (anti-CCP antibody), and anti-neutrophil cytoplasmic antibodies (ANCA). Urine and blood cultures results were negative, as well as interferon gamma release assay (Quantiferon).

A chest radiograph disclosed mild interstitial thickening, and multiple osteoporotic fractures of the D7, D8, and D11 vertebral bodies were present on spinal X-rays. Hand, wrist and knee radiographs demonstrated alterations consistent with osteoarthritis without joint erosions.

**Fig. 1:**
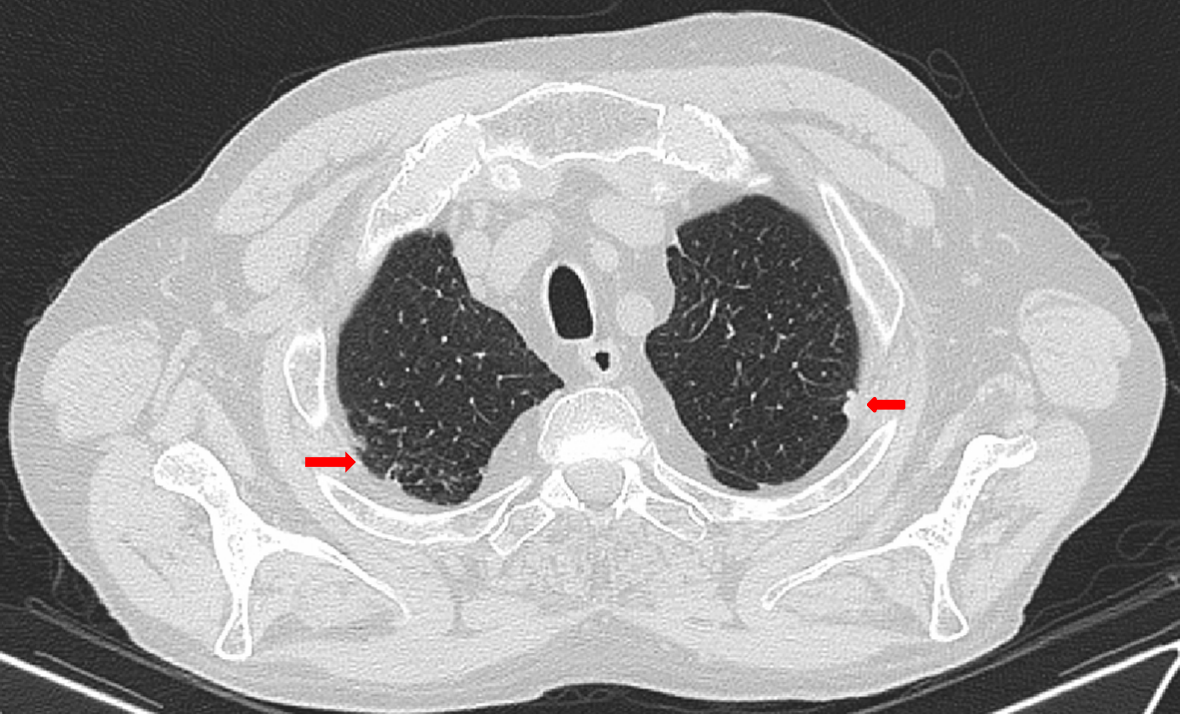
Thoracic HRCT. Bilateral subpleural fibrosis with pleural thickening and presence of plaques (arrows) suggesting asbestosis

**Fig. 2:**
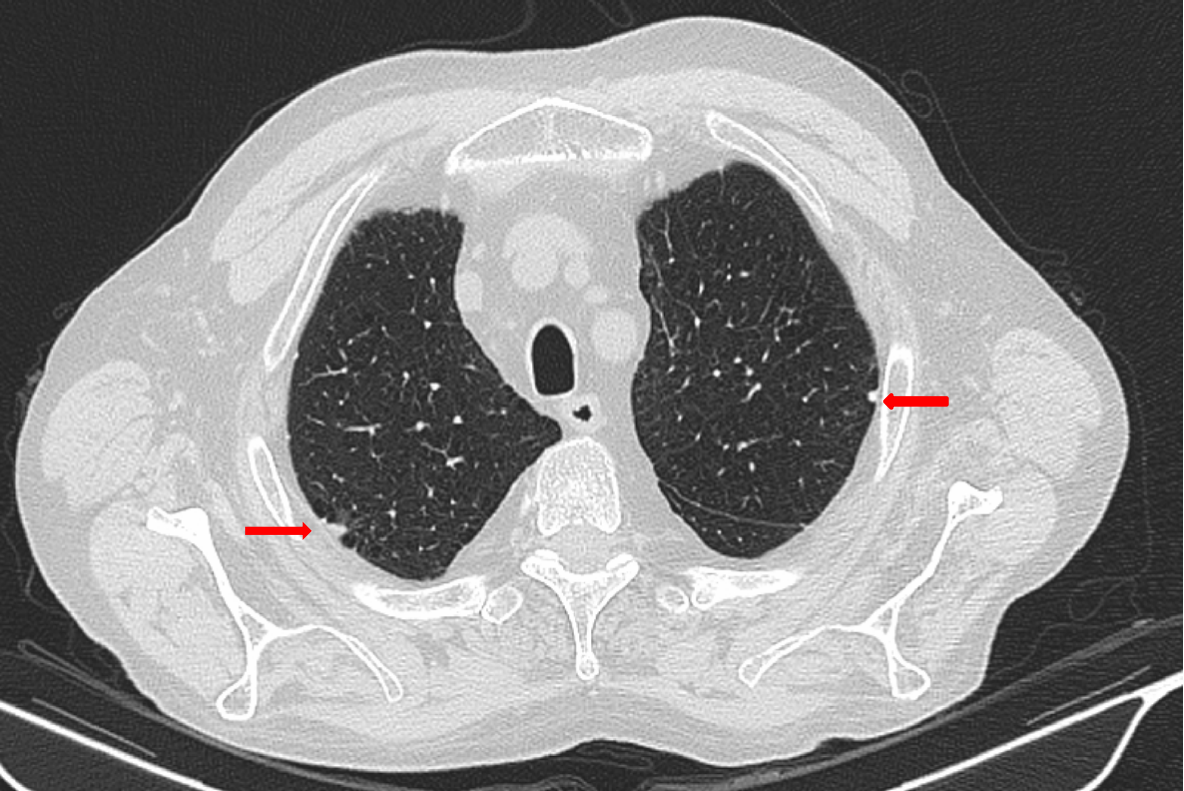
Thoracic HRCT. Presence of pleural plaques

**Fig. 3:**
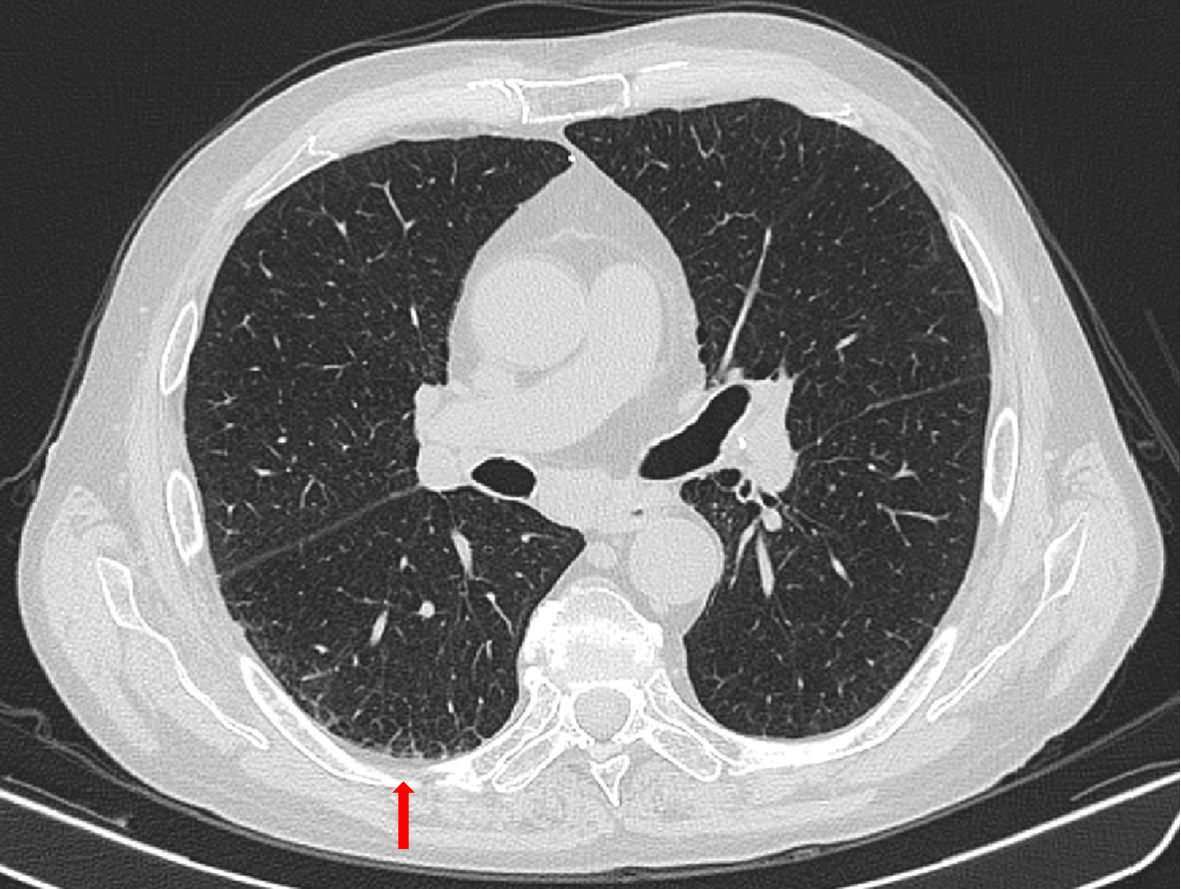
Thoracic HRCT. Pleural thickening and fibrosis

HRCT of the lung showed pulmonary fibrosis with pleural plaques suggesting asbestosis (Figs. 1, 2 and 3).

Based on the patient’s history of asbestos exposure, clinical, laboratory and instrumental investigations, a diagnosis of mild asbestosis with prominent asbestos-induced systemic autoimmune disease features was made, and, considering the contraindication to corticosteroid use due to the severe osteoporotic vertebral fractures, and the recent data reported in the literature showing a pathogenic role of IL-1 beta in pneumoconiosis, subcutaneous canakinumab at a dose of 150 mg every 8 weeks was proposed. After obtaining local ethical Committee approval and the patient’s written informed consent, canakinumab was started. The patient was scheduled to receive the injections of canakinumab in the outpatient office of our rheumatology division.

At the 7-day control visit, the patient was asymptomatic and his ESR and CRP had declined to 36 mm/h and 1.6 mg/dl, respectively. Canakinumab was continued and after 4 months of follow-up the patient was still in clinical remission and laboratory examination demonstrated normal acute-phase reactants (ESR: 12 mm/h; CRP: 0.19 mg/dl) and a diminished ANA titer of 1/320. The therapy was well tolerated without adverse events or side effects.

## Discussion

Systemic autoimmune disease in patients with asbestosis has been reported in terms of both autoantibody production and systemic autoimmune clinical manifestations. Investigations on several cohorts of asbestos-exposed subjects have demonstrated either antinuclear antibody positivity in 1.5 to 70 % and RF positivity in 1.4 to 35 % [7]. This rather wide range of reported autoantibody positivity may be in part explained by the different types of inhaled asbestos dust, as demonstrated by experimental studies in mice showing that amphibole, but not chrysotile, asbestos induces an autoimmune response, although both fibers activate the NALP3 inflammasome [11]. The different immunogenicity of the two fibers seems to be related to distinct inflammatory pathway activation by the amphibole and chrysotile fibers [11].

RA represents the most frequent systemic autoimmune disease associated with asbestos exposure, and clinical findings of RA in patients with asbestosis were first described more than 40 years ago [5, 12]. An increased risk of RA in asbestos-exposed subjects (Odds ratio [OR] 2.5 [95 % CI, 1.0–6.8]) was reported in a Swedish study [13], and in a case-control study, the asbestos-exposed population of Libby, Montana, was compared to the unexposed population of Missoula, Montana. A significantly increased frequency of systemic autoimmune disease (OR 2.14 [95 % CI, 0.90–5.10]), and a higher risk of RA (OR 3.23 [95 % CI, 1.31-7.96]) were observed in the exposed population [6]. In the same study, an association between asbestos exposure and SLE was found, in keeping with the previously reported high frequency of ANA positivity in the Libby population [4]. However, apart from single case reports [14], other population-based studies evaluating the risk of SLE in asbestos-exposed workers are lacking.

Other systemic autoimmune diseases, including systemic sclerosis, ANCA-associated vasculitis, and retroperitoneal fibrosis, have been reported sporadically in patients with asbestosis [7].

As described, our patient had a history of occupational exposure and typical clinical and radiological findings of asbestosis with associated persistent ANA positivity, low grade fever, arthralgia, and episodes of mild arthritis without evidence of articular erosions, and was negative for RF and anti-CCP antibody. Hence, he met the criteria for UCTD [15] rather than RA, or, as proposed by other Authors [6], he had systemic autoimmune disease associated with asbestosis.

Over the last decades, experimental studies have shown the crucial role exerted by IL-1 in pulmonary homeostasis and pathology [16, 17]. The IL-1 family is composed by IL-1alpha and IL-1beta, two major agonistic molecules, and IL-1R antagonist (IL-1Ra) that bind to the same IL-1 receptor [18]. IL-1alpha precursor, which is present intracellularly in healthy tissues and during hypoxic death (but not in apoptosis), is released from cells undergoing necrosis and is biologically active, while IL-1beta precursor is produced be monocytes and macrophages, and it is activated after cleavage by caspase 1 which in turn is activated by NALP3 inflammasome [18]. Regarding the lung, experimental studies have shown that in absence of inflammatory stimuli, alveolar type II cells enhance the production of prostaglandin E2 which in turn inhibits fibroblast proliferation through an IL-1 alpha mediated pathway [19]. Under inflammatory stimuli such as silica or asbestos inhalation, IL-1 beta seems to play a pivotal role in the pathogenesis of fibrosis and mesothelioma [10]. Indeed, inhaled silica or asbestos are captured by macrophages with activation of NALP3 inflammasome which induces the conversion of procaspase 1 in an active form to cleave the IL-1 beta precursor in active IL-1beta with consequent fibrotic nodules formation [10, 20]. IL-1beta is also involved in T-lymphocyte activation with subsequent dysregulated autoimmunity and autoantibody production [3]. An increased release of IL-1beta by alveolar macrophages in patients with asbestosis was reported around 20 years ago [16], and increased serum levels have been found in coal workers with pneumoconiosis and in cement mason apprentices [21, 22].

Based on the consistent body of evidence of the pathogenic role of inflammasome-dependent release of IL-1beta in patients with asbestosis and related systemic autoimmune disease, we decided to treat our patient with canakinumab, a specifically targeted anti-IL-1beta antibody, which has been licensed for the treatment of inflammasome-mediated autoinflammatory syndromes [23]. The dramatic improvement of clinical features and acute phase reactants over 1 week encouraged us to continue the treatment, and, at the 4-month visit, the patient achieved clinical remission. Similar favorable results have been recently reported in a patient with silicosis treated with anakinra, an IL-1 receptor antagonist acting on both IL1 alpha and Il-1 beta [24]. Differently from this report, given the central pathogenic role of IL-1 beta, we decided to employ canakinumab due to its selective action directed against this cytokine, and, according to patient’s preference, for its lower frequency of administration.

## Conclusion

The successful treatment of asbestosis-related systemic autoimmune disease with canakinumab observed in our patient seems to confirm the reported pivotal role exerted by inflammasome and IL-1 beta in this clinical condition. Our case does not provide indication of the effects of anti-IL-1 beta targeted therapy on the progression of lung fibrosis, and does not allow definitive conclusions to be drawn, but may suggest a new perspective for the treatment of systemic autoimmune features of asbestosis and, possibly, of lung involvement.

### Patient’s consent

A written informed consent was obtained from the patient for publication of this case report and any accompanying images. A copy of the written consent is available for review by the Editor of this Journal.

## Abbreviations

HRCT: High resolution computed tomography
ANA: Serum antinuclear antibody
RF: Rheumatoid factor
SLE: Systemic lupus erythematosus
RA: Rheumatoid arthritis
IL-1beta: Interleukin 1-beta
ESR: Erythrocyte sedimentation rate
CRP: C-reactive protein
UCTD: Undifferentiated connective tissue disease
anti-CCP: Anti-cyclic citrullinated peptide antibody
ANCA: Anti-neutrophil cytoplasmic antibodies
